# AGE DIFFERENCES IN SOCIOCULTURAL DETERMINANTS OF INDIGENOUS HEALTH

**DOI:** 10.1093/geroni/igae098.4115

**Published:** 2024-12-31

**Authors:** Amanda Goldtooth, Raechel Livingston, Eric Cerino

**Affiliations:** Northern Arizona University, Flagstaff, Arizona, United States; Northern Arizona University, Flagstaff, Arizona, United States; Northern Arizona University, Flagstaff, Arizona, United States

## Abstract

Healthy aging frameworks often lack a culturally-informed theoretical approach to investigate how sociocultural resources advance health equity, particularly for Indigenous adults. Our work addressed this gap by empirically testing Yellow Bird and colleagues’ (2023) Cultural Determinants of Healthy Indigenous Aging model. Specifically, we examined age differences in associations between sociocultural resources and health. In a sample of 85 Native American or Alaska Native adults from the Midlife in the United States study (average age=54.25 years, SD=12.86, 52% female), respondents completed surveys on sociocultural resources (spirituality, neighborhood quality, purpose) and self-reported health outcomes (physical health, negative affect, positive affect). We regressed each health outcome on the sociocultural resources and adjusted for age, sex, and education. We also examined whether age moderated these associations. People with higher purpose reported better self-rated physical health (Est.=0.06, SE=0.01, p<.001), lower negative affect (Est.=-0.05, SE=0.01, p<.001), and higher positive affect (Est.=0.04, SE=0.01, p<.001). Age moderated the relationship between spirituality and positive affect (Est.=0.01, SE=0.002, p<.01) such that higher spirituality related to higher positive affect among comparatively older adults (Est.=0.14, SE=0.04, p<.01). People with better neighborhood quality reported better self-rated physical health (Est.=0.61, SE=0.20, p<.01). Age moderated the relationship between neighborhood quality and positive affect (Est.=0.02, SE=0.01, p<.05) such that better neighborhood quality related to higher positive affect among comparatively older adults (Est.=0.48, SE=0.19, p<.05). Consistent with Yellow Bird and colleague’s (2023) culturally-informed framework, results suggest that purpose, neighborhood quality, and spirituality may serve as sociocultural pathways to promote healthy Indigenous aging and advance health equity.

